# Effect of the ZiBuPiYin Recipe on Diabetes-Associated Cognitive Decline in Zucker Diabetic Fatty Rats After Chronic Psychological Stress

**DOI:** 10.3389/fpsyt.2020.00272

**Published:** 2020-04-21

**Authors:** Tingting Bi, Libin Zhan, Wen Zhou, Hua Sui

**Affiliations:** ^1^ Modern Research Laboratory of Spleen Visceral Manifestations Theory, School of Traditional Chinese Medicine & School of Integrated Chinese and Western Medicine, Nanjing University of Chinese Medicine, Nanjing, China; ^2^ Institute of Integrative Medicine, Dalian Medical University, Dalian, China

**Keywords:** diabetes-associated cognitive decline, chronic psychological stress, ZiBuPiYin recipe, *β*-amyloid, IRS-1/AKT/mTOR signaling pathway

## Abstract

**Background:**

Cognitive impairment is a complication of type 2 diabetes mellitus (T2DM) that affects the central nervous system (CNS). Studies have shown that chronic psychological stress may promote the development of T2DM into diabetes-associated cognitive decline (DACD). Previously, cognitive impairment in T2DM was correlated predominantly with insulin resistance in the medial prefrontal cortex (mPFC).

**Aims:**

We examined the effect of the ZiBuPiYin recipe (ZBPYR) on Zucker diabetic fatty (ZDF) rats and explored the impact of chronic stress on altered *β*-amyloid (A*β*) metabolism through insulin receptor substrate (IRS) 1/protein kinase B (AKT)/mammalian target of rapamycin (mTOR) signaling pathway after the induction of chronic psychological stress.

**Main Methods:**

After chronic psychological stress and drug treatment, cognitive function was observed *via* behavioral experiments. The activation of the hypothalamus-pituitary-adrenal (HPA) axis and levels of A*β* were detected by enzyme-linked immunosorbent assay, and the expression of related proteins was evaluated by Western blotting.

**Key Findings:**

ZBPYR treatment significantly decreased anxious-like behaviors and plasma corticosterone (CORT) levels, and ameliorated learning and memory impairments of ZDF rats after chronic psychological stress. ZBPYR also reduced the deposition of A*β* in the mPFC, improved brain insulin resistance, and modulated the mTOR-autophagy pathway.

**Significance:**

ZBPYR may be a potential therapeutic application for the treatment of DACD induced by chronic psychological stress.

## Introduction

Type 2 diabetes mellitus (T2DM) is a metabolic disorder characterized by chronic hyperglycemia, increased insulin resistance over time, and progressive failure of pancreatic insulin secretion (1). Epidemiologic studies have reported that the worldwide prevalence of diabetes mellitus is expected to increase from 425 million in 2017 to an estimated 629 million in 2045 (2). Both cross-sectional and longitudinal studies reported that T2DM is associated with Alzheimer's disease (AD) (3), mild cognitive impairment (MCI) (4), anxiety, and depression (5). Diabetes-associated cognitive decline (DACD), a series of slight cognitive decrements over time, is generally thought to be a central nervous system (CNS) complication of T2DM (6, 7).

The development and progression of DACD is a complex process that likely involves synergistic effects of genetic susceptibility and environmental factors (8, 9). Previous studies have shown both cerebral insulin resistance and altered *β*-amyloid (A*β*) metabolism could affect cognitive function of DACD (10–12). In addition, insulin receptor substrate (IRS) 1/protein kinase B (AKT)/mammalian target of rapamycin (mTOR) pathways play a critical role in insulin signaling. Insulin recruits and activates the IRS and activates the downstream AKT cascade, which then activates the mTOR-autophagy pathway and affects A*β* deposition. Hence, interference with the IRS1/AKT/mTOR pathway may lead to cognitive impairment.

Recent reports have shown that changes in lifestyle could increase the risk for DACD (13, 14). In particular, there is persuasive evidence that psychological stress contributes to both T2DM and DACD (14). Although immediate psychological stress is not considered an issue, chronic activation of psychological stress is a potent pathogenic factor in these disorders (15). Chronic psychological stress makes it increasingly difficult for the body to function, which not only impacts mental health, but can also predispose a person to various metabolic disorders (16, 17). Thus far, the molecular, physiological, and behavioral pathways involved in chronic psychological stress-induced DACD and the link between chronic psychological stress and changes in the brain that accompany DACD have not been clarified. Because chronic psychological stress adversely influences quality of life T2DM patients, developing a strategy to avoid DACD is important.

ZiBuPiYin recipe (ZBPYR), a traditional formula of Chinese medicine documented in the book of Bujuji written by Wu Cheng in the Qing dynasty, is derived from Zicheng Decoction and used for the treatment of cognitive impairment. Early reports have shown that ZBPYR improves the learning and memory process in high-fat diet combined with low-dose streptozotocin (STZ)-induced diabetic rats, and regulates the deposition of A*β* in the brain (18). Our recent data also demonstrated that chronic psychological stress impairs glucose intolerance and decreases insulin sensitivity in Zucker diabetic fatty (ZDF) rats, suggesting that chronic psychological stress can contribute to the development of insulin resistance in T2DM (19). In the present work, we used spontaneous ZDF rats as a T2DM model and examined the hypothesis that ZBPYR could reverse the impairment of chronic psychological stress leading to DACD, mainly by reducing A*β* deposition through the IRS-1/AKT/mTOR signaling pathways in the medial prefrontal cortex (mPFC).

## Materials and Methods

### Reagents and Antibodies

Antibody against amyloid-*β* protein precursor (APP) was acquired from the Sigma-Aldrich (St Louis, MO, USA). The Inhibitors Protease Inhibitor Cocktail and Phosphatase Inhibitor Cocktail and the antibodies against Phospho-IRS1 (Ser307), AKT, phospho-AKT (Ser473), Phospho-mTOR (Ser2448), phospho-P70S6K (Thr389), LC3A/B, *β*-actin and secondary antibody horseradish peroxidase (HRP)-conjugated goat-anti-mouse IgG, and goat-anti-rabbit IgG were obtained from Cell Signaling Technology (Beverly, MA, USA). Antibodies against IRS-1, GSK3*β*, Phospho-GSK3*β* (Ser9), mTOR, and P70S6K were acquired from Abcam (Cambridge, MA, USA). Western blotting material was obtained from Bio-Rad (Hercules, CA, USA). All other experimental supplies and reagents were purchased from Biosharp (Shanghai, China) and Solarbio (Beijing, China).

### Animal Model

Male 6-week-old ZDF (*fa/fa*) rats were purchased from Vital River Laboratories (VRL) (Beijing, China) and housed in a specific pathogen-free (SPF) animal experiment center at Nanjing University of Chinese Medicine. The ZDF strain has a homozygous leptin receptor mutation pre-disposing the rats to T2DM. When inbred ZDF males are fed the Purina 5008 diet (protein = 23.6%, nitrogen-free extract [by difference] = 50.3%, fiber [crude] = 3.3%, ash = 6.1%, fat [ether extract] = 6.7%, and fat [acid hydrolysis] = 8.1%; Vital River Laboratories, Beijing, China], which is high in carbohydrates and fats, they exhibit hyperinsulinemia, hyperglycemia, hypercholesterolemia, and hypertriglyceridemia, mimicking a type 2 diabetic state.

All rats were provided food and water ad libitum. They were maintained at 23°C ± 2°C with 65% ± 5% humidity on a 12-h light/dark cycle and were allowed to acclimatize to their environment for one week prior to drug or ultrapure water administration. All animal experiments were conducted in accordance with the National Institutes of Health Guide for the Care and Use of Laboratory Animals at Nanjing University of Chinese Medicine (Nanjing, China), and were approved by the Animal Ethics Committee of Nanjing University of Chinese Medicine (approval no. ACU170606).

### Preparation and Administration of ZBPYR

ZBPYR were purchased from the Sanyue Chinese Traditional Medicine Co., Ltd. (Nantong, China), as shown in **Table 1**. Voucher specimens were certified by the company, and quality inspection reports were provided. The administration and the exact compounds/chemicals of ZBPYR were provided in previous studies (20, 21): the mixtures were soaked in 8 volume per weight (1:8, w/v) of distilled water for 30 min and subsequently boiled for 90 min, then Tan-Xiang was added and boiled for 30 min. The mixtures were extracted twice. The decoction was ﬁltered and concentrated to a final density of 3.29 g/ml.

**Table 1:** The ingredients of ZBPYR.

Herbal name	Botanical Latin name	Place of origin	Part used	Amount used (g)
Hong-Shen	*Panax ginseng* C. A. Mey.	Jilin	Root	30
Shan-Yao	*Dioscorea polystachya* Turcz.	Henan	Rhizome	15
Fu-Shen	*Poria cocos* (Schw.) Wolf	Anhui	Root	15
Bai-Shao	*Paeonia lactiflora* Pall.	Anhui	Root	15
Dan-Shen	*Salvia miltiorrhiza* Bunge	Shandong	Root	12
Bai-Bian-Dou	*Lablab purpureus* (L.) Sweet	Zhejiang	Bean	15
Lian-Zi	*Nelumbo nucifera* Gaertn.	Hunan	Seed	20
Shi-Chang-Pu	*Acorus gramineus* Sol. ex Aiton	Zhejiang	Rhizome	10
Yuan-Zhi	*Polygala tenuifolia* Willd.	Hebei	Root	10
Tan-Xiang	*Santalum album* L.	Guangdong	Sandalwood	4.5
Ju-Hong	*Citrus maxima* “Tomentosa”	Sichuan	Epicarp	9
Gan-Cao	*Glycyrrhiza uralensis* Fisch. ex DC.	Neimenggu	Root	9
**Total amount**				164.5

### Experimental Design

After 1 week of adaptive feeding, rats were randomly divided into 3 groups (n=6 each): ZDF model (ZDF group), chronic psychological stress-induced DACD (PSD group), and chronic psychological stress combined with ZBPYR administration (PDZ group). The PSD group and PDZ groups were subjected to two stress simulations for 6 weeks: restriction and rotation. During the restriction simulation, rats were put in open bottles. The size of the bottle did not allow the rat to turn around. The experimental time was limited to 2 h and was employed every other day. In the rotation simulation, the rats were placed in the homemade rotating device rotating for 15 min at 30 rpm with an interval ranging from 40 to 150 min. Four cycles were performed in each experiment every other day. It should be noted that rotation stress and restriction stress tests were not carried out on the same day. During the stress simulations, ZDF group simultaneous removal food and water.

**Figure 1:**
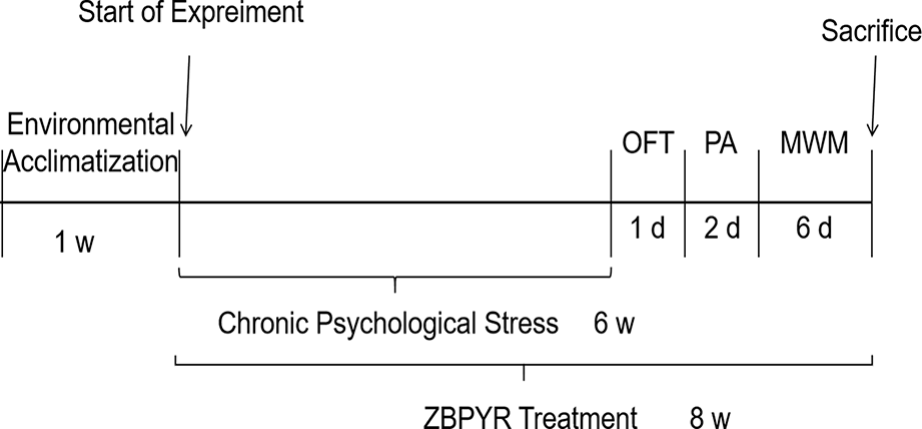
Experimental process. OFT, open field test; PA, passive avoidance test; MWM, the Morris water maze test.

During a period of 8 weeks, ZBPYR was administered by oral gavage daily at a dose of 32.9 g/kg body weight in the PDZ group, while other groups were orally administered an equal dose of ultrapure water instead of ZBPYR (Milli-Q Integral Water Purification System, Millipore Corporation, Billerica, MA, USA). The experimental process is shown in **Figure 1**.

### Measurement of Plasma Corticosterone Levels

We measured plasma corticosterone (CORT) levels to determine whether the hypothalamic pituitary adrenal (HPA) axis was activated. After the end of chronic psychological stress, 100 µL of blood was collected from the tail vein of each rat. Blood samples were centrifuged at 1,000 × g for 10 min at 4°C. The supernatant plasma was collected and stored at −80°C until required. The concentration of CORT levels in plasma were measured using enzyme-linked immunosorbent assay (ELISA) kits according to the manufacturer's instructions and calculated based on the standard curve.

### Behavioral Tests

Behavioral tests, including an open field test (OFT), a passive avoidance (PA) test, and the Morris water maze test (MWM), were performed during the last 9 days of treatment, according to the following schedule: day 1, OFT; days 2–3, PA test; and days 4–9 MWM test.

#### OFT

The OFT assessed the effects of chronic psychological stress on anxious behavior. Briefly, rats were placed in the test environment for 1 h prior to testing. After the start of the test, rats were placed in the center of a dimly illuminated open field chamber (50 cm × 50 cm × 50 cm) and allowed to explore freely for 5 min. The square arena was divided into a center zone (10 cm × 10 cm) and an outer zone. Behavioral patterns measured in the chamber included time in the center %, entries in the center, total distance, and velocity. All animals were used only once, and the locomotion was recorded by video camera and registered in the computer. After each trial, the square arena was cleaned with 70% alcohol to prevent the influence of the odors present in the urine and feces of the previous rat.

#### PA

In the PA test, animals learned to avoid an environment in which an adverse stimulus (foot shock) was applied. The PA apparatus consisted of two identical compartments separated by a guillotine opaque door. One compartment was illuminated (safe), and the other was dark. The procedure was done on two consecutive days. On the first day, animals were individually placed into the safe compartment, allowed to freely explore for 5 s, and allowed to move into the dark compartment by opening the door. Training was done by closing the door and applying a mild electrical foot shock (1 mA for 5 s) upon entry of the rat into the dark compartment. Memory test was conducted 24 h after training. The procedure was performed the same as training without any shock. Each rat was again positioned in the safe compartment backing to the door. The time to enter the dark compartment was recorded as the error number. The latency period to enter the dark compartment was acquired during a maximum of 300 s.

#### MWM

Spatial learning and memory performance of the rats were evaluated by the MWM test, which consisted of 5 days of training (invisible plat training sessions and a probe trial), and a visible platform training session on day 6. The apparatus consisted of a circular pool (160 cm in diameter, 50 cm in height, and filled to a depth of 40 cm with water maintained at 24°C ± 1°C) and a platform (12 cm in diameter and 29 cm in height), which was hidden under water. On the first day, rats were permitted to swim freely in the tank for 120 s without the platform to adapt to the new condition. Over the following 4 days, rats were trained with 4 trials per day at intervals of 120 s until they reached the platform and were allowed to rest for 60 s. If the rat failed to reach the platform within 120 s, it was gently guided to the platform and allowed to stay for 60 s. The escape latency taken to reach the platform was measured automatically. For the probe trial test, the platform was removed. Rats had to swim for 120 s, and time searching for the original platform and time across the platform area were recorded. On day 6, a visible platform test was performed. The platform was located 2 cm over the water surface and placed in a position different from the previous test. During this test, the escape latency was recorded. All data were measured by a camera and automated analysis system.

### Brain Sample Collection

Rats were deeply anesthetized with isoflurane and decapitated. Brains were removed, dissected, and the mPFC was isolated on ice and rapidly frozen in liquid nitrogen. All dissociated tissues were stored at −80°C until required.

### ELISA Analyses

Frozen mPFC samples were homogenized in liquid nitrogen and extracted with buffer (1× TBS containing 5 mM EDTA and 2 mM 1,10-phenanthroline). The homogenates were kept at 4°C for 15 min and then centrifuged at 500,000×*g* for 1 h at 4°C. The supernatants were collected, and soluble A*β*40 and A*β*42 levels were measured using ELISA kits. Afterward, the pellets were homogenized in 70% formic acid (FA) solutions and centrifuged at 500,000×*g* for 1 h at 4°C. The FA-containing supernatants were neutralized *via* a 1:20 dilution into 1 M Tris-base (pH=11), and insoluble A*β*40 and A*β*42 levels were then measured using ELISA kits. All experiments were performed per the manufacturer's instructions. The optical density of each well was read at 450 nm using a microplate spectrophotometer.

### Western Blotting

Frozen mPFC samples were lysed for 30 min in an ice-cold radioimmunoprecipitation assay (RIPA) buffer (Beyotime, Shanghai, China) containing 100× Inhibitors Protease Inhibitor Cocktail and 100× Phosphatase Inhibitor Cocktail to extract total proteins. Following centrifugation (14,800 rpm, 10 min), the supernatants were harvested, and protein concentrations were measured using a Minim Spectrophotometer. Subsequent to boiling and denaturing, the same amounts of protein samples (25 μg extract/lane) were loaded and separated by 8% sodium dodecylsulfate-polyacrylamide gel electrophoresis. Then the proteins were electro-transferred onto polyvinylidene difluoride membranes (Millipore, Bedford, MA, USA). After blocking with 5% non-fat milk in Tris-buffered saline Tween-20 (TBST, 50 mM Tris-HCl, 150 mM NaCl, pH 7.4, 0.1% Tween-20) for 2 h at room temperature, the membranes were incubated overnight with targeted primary antibodies in TBST at 4°C as follows: p-IRS1 (1:1000), IRS1 (1:1000), p-AKT (1:1000), AKT (1:1000), p-GSK3*β* (1:1000), GSK3*β* (1:1000), p-mTOR (1:1000), mTOR (1:1000), p-P70S6K (1:1000), P70S6K (1:1000), and LC3A/B (1:1000). After washing, the membranes were incubated for 1 h at room temperature with peroxidase-conjugated secondary antibodies. After washing with TBST 3 times for 5 min, the membranes were incubated with secondary antibodies conjugated to HRP (1:2000) for 60 min at room temperature. The washings were then repeated. For visualization, the immunoreactive bands were treated with a chemiluminescence solution (ECL, Tanon, Shanghai, China) and detected with X-ray films. The optical density values of the target protein bands were quantified with ImageQuant TL 1D (GE Healthcare, USA) and normalized to *β*-actin (1:1000) loading control. The results were expressed as the means of three experiments.

### Statistical Analysis

All data were obtained from at least three independent experiments and expressed as means ± standard error of means (SEM). Statistical significance was analyzed using one-way analysis of variance (ANOVA) followed by Tukey's *post hoc* test or Fisher's LSD. All statistical analyses were performed using SPSS 19.0 (IBM, Chicago, IL, USA). *P* values < 0.05 were considered significant.

## Results

### Impact of ZBPYR on Anxious-Like Behaviors and Plasma CORT Levels

**Figure 2:**
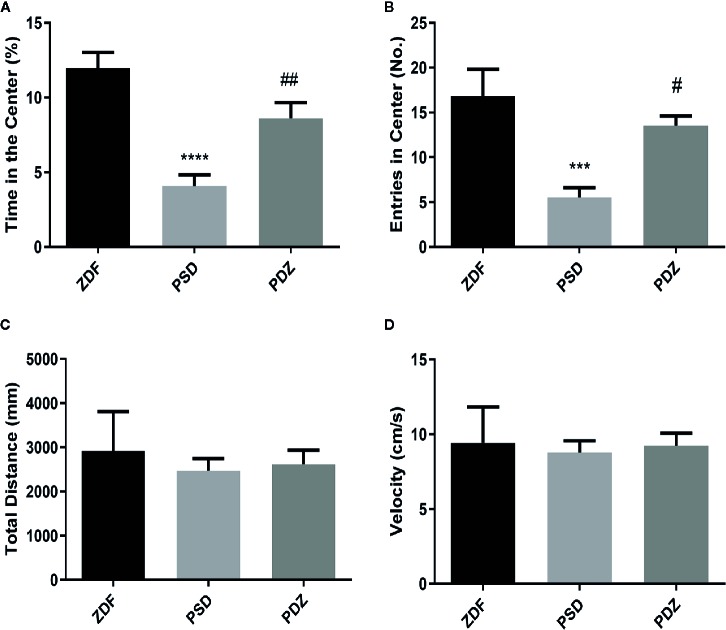
Effect ZBPYR on activities in the open-field test. Performance of ZDF rats in each group was measured, including time in the center (%) **(A)**, entries in the center **(B)**, total distance **(C)** and velocity **(D)**. Bars represent the mean ± SEM of 6 rats per group. ^***^ *P* < 0.001, ^****^ *P* < 0.0001, PSD group *vs.* ZDF group; ^#^ *P* < 0.05, ^##^ *P* < 0.01, PDZ group *vs.* PSD group.

Rats exposed to chronic psychological stress exhibited a series of abnormal behaviors, such as piloerection, indolence, and fidgeting, which were typical anxiety-like behaviors. In the OFT, the PSD group was in the center for a shorter time (**Figure 2A**; *P* < 0.01) and entered the center less frequently (**Figure 2B**; *P* < 0.01) compared with ZDF group. The behavior of rats in the PDZ group returned to normal levels after 8 weeks of treatment of ZBPYR. ZDF rats gained weight from 8 to 15 weeks of age (22), but the behavioral experiments involved in our study did not find an effect of body weight on the experimental results, as shown by the total distance and velocity of movement of rats. There were no significant changes in physical activity in the three groups (**Figures 2C, D**).

**Figure 3:**
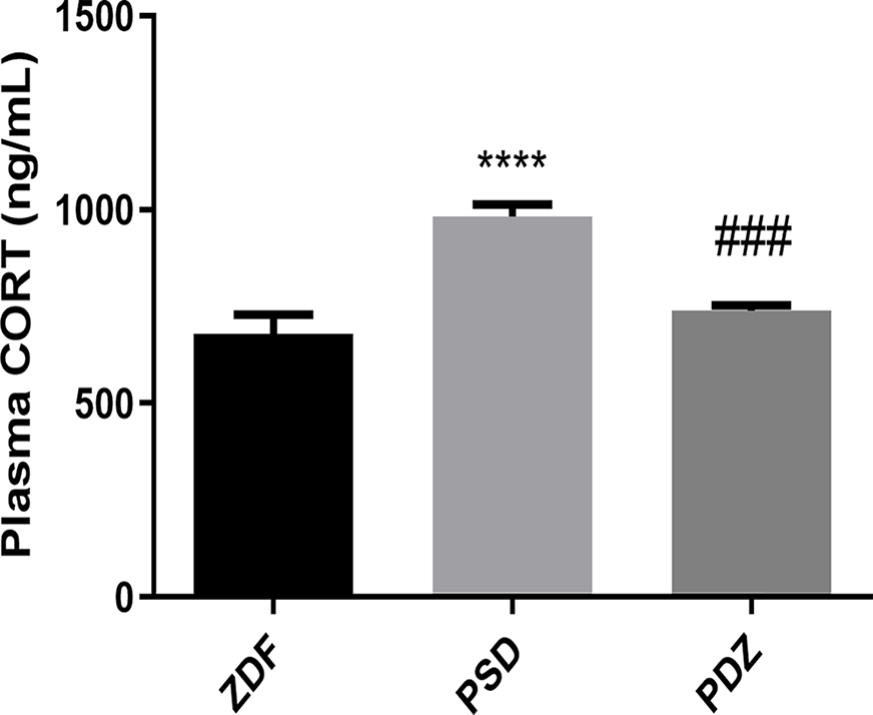
Effect of ZBPYR on plasma corticosterone (CORT) levels. Bars represent the mean ± SEM of 6 rats per group. ^****^ *P* < 0.0001, PSD group *vs.* ZDF group; ^###^ *P* < 0.001, PDZ group *vs.* PSD group.

We measured plasma CORT hormone levels in all rats to determine whether the HPA axis was activated CORT levels in the PSD group were significantly higher than in the ZDF group, while the groups that received ZBPYR showed significantly decreased CORT levels (**Figure 3**).

### Impact of ZBPYR on Cognitive Impairment

**Figure 4:**
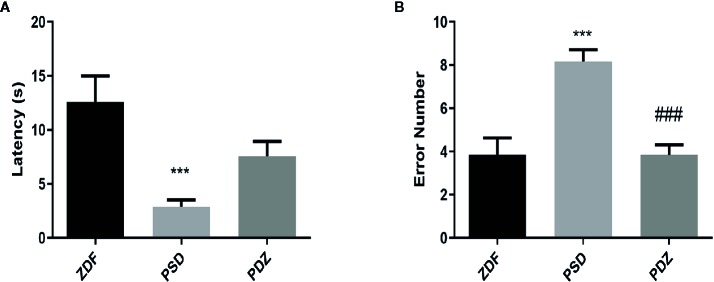
Effect of ZBPYR on cognitive impairment in the passive avoidance test. The latency **(A)** and error number **(B)** were assessed. Bars represent the mean ± SEM of 6 rats per group. ^***^ *P* < 0.001, PSD group *vs.* ZDF group; ^###^ *P* < 0.001, PDZ group *vs.* PSD group.

**Figure 5:**
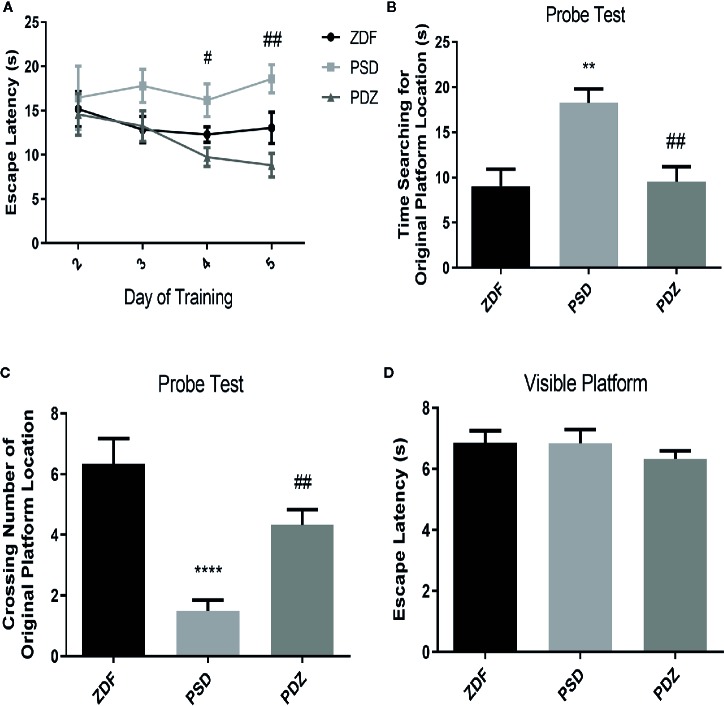
Effect of ZBPYR on cognitive impairment in the Morris water maze (MWM) test. The escape latencies of the animals were analyzed in the training trials **(A)**. Performance in the probe test was analyzed, including time searching for the original platform location **(B)** and number of crossings over the original platform location **(C)**. Escape latency in the visible platform version of the MWM test was also analyzed **(D)**. Bars represent the mean ± SEM of 6 rats per group. ^**^ *P* < 0.01, ^****^ *P* < 0.0001, PSD group *vs.* ZDF group; ^#^ *P* < 0.05, ^##^ *P* < 0.01, PDZ group *vs.* PSD group.

To investigate whether chronic psychological stress affects cognitive function, we evaluated the passive avoidance memory and spatial learning abilities in different groups by PA test and MWM test. In the PA test, the PSD group had a significantly lower latency and a greater error number than the ZDF group. However, cognitive impairment was significantly reversed after 8 weeks of ZBPYR administration (**Figures 4A, B**; *P* < 0.05; *P* < 0.01). MWM results showed that the time to find the hidden platform progressively decreased in the PDZ group (**Figure 5A**, *P* < 0.05). In the invisible plat training test, the escape latency of the PSD group was significantly longer than that of the ZDF group. In contrast, the PDZ group showed a shorter escape latency compared with PSD group. In the probe trial test, we found that the PSD group cost more seconds during the test, and number of times across the original platform area were significantly lower than that of ZDF group (**Figures 5B, C**, *P* < 0.05; *P* < 0.01). Performance in the visible platform version was similar across groups in terms of escape latency (**Figure 5D**). All these findings suggest that ZBPYR could attenuate the impairment of learning and memory impairments caused by chronic psychological stress in ZDF rats.

### Impact of ZBPYR on Aβ Deposition

**Figure 6:**
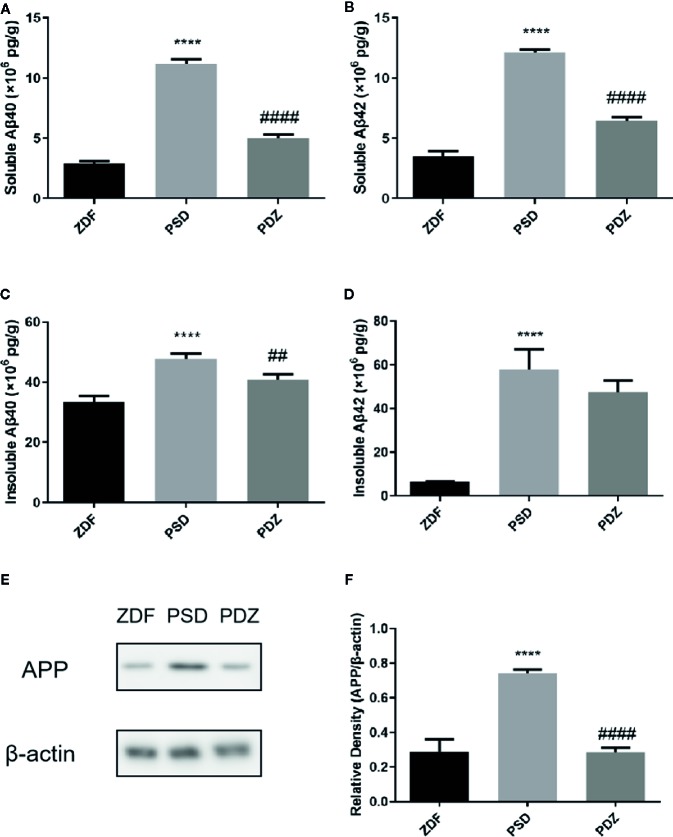
Effect of ZBPYR on generation and deposition of *β*-amyloid in the medial prefrontal cortex (mPFC) of ZDF rats. Levels of soluble and insoluble A*β*40 and A*β*42 in the mPFC of each group were measured using enzyme-linked immunosorbent assay **(A–D)**. Protein expression of APP in the mPFC of each group were detected by western blotting **(E, F)**. Bars represent the mean ± SEM. ****P < 0.0001, PSD group vs. ZDF group; ^##^ *P* < 0.01, ^####^ *P* < 0.0001, PDZ group vs. PSD group.

A*β* is the main component of amyloid plaques. To determine whether cognitive impairments were associated with A*β* deposition, we assessed both soluble and insoluble A*β*40 and A*β*42 levels in the mPFC of ZDF rats. The soluble A*β*40 and A*β*42 levels in the PSD group were significantly higher compared with the ZDF group, whereas in the PDZ group, levels were further downregulated after ZBPYR-treatment (**Figures 6A, B**, *P* < 0.001). Similarly, the insoluble A*β*40 and A*β*42 levels in the PSD group were significantly increased compared with the ZDF group, whereas the insoluble A*β*42 levels were not affected by ZBPYR in the PDZ group (**Figures 6C, D**, *P* < 0.001).

We further detected the expression patterns of APP, where cerebral A*β* production originates. Consistent with the ELISA results, the protein expression levels of APP were markedly significantly increased in the PSD group, and reduced after administration of ZBPYR (**Figures 6E, F**, *P* < 0.001), indicating that ZBPYR reduces A*β* generation and deposition in ZDF rats after chronic psychological stress.

### Impact of ZBPYR on Brain Insulin Resistance

**Figure 7:**
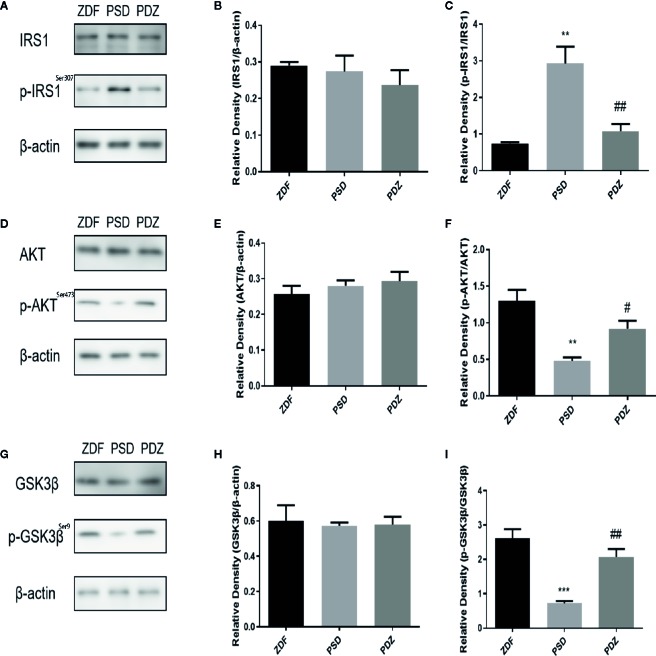
Effect of ZBPYR on cerebral insulin signaling and GSK3*β* in the mPFC of ZDF rats. Protein expression of IRS1 and p-IRS1 in the mPFC of each group **(A–C)**. Protein expression of AKT and p-AKT in the mPFC of each group **(D–F)**. Protein expression of GSK3*β* and p-GSK3*β* in the mPFC of each group **(G–I)**. Bars represent the mean ± SEM. ^**^ *P* < 0.01, ^***^ *P* < 0.001, PSD group *vs.* ZDF group; ^#^ *P* < 0.05, ^##^ *P* < 0.01, PDZ group *vs.* PSD group.

The IRS1/AKT/GSK-3*β* pathway was pivotal to maintenance of the neuronal network and cognitive ability. To evaluate the effect of ZBPYR on insulin signaling in ZDF rats after chronic psychological stress, we detected the expression of insulin signaling factors IRS1 and p-IRS1, AKT, and p-AKT in mPFC by western blotting analysis. No significant changes in total levels of IRS1 and AKT were seen in the three groups (**Figures 7B, E**). However, we observed a significant increase in p-IRS1 levels and a significant down-regulation of p-AKT levels in the PSD group. In the PDZ group, the expression of p-IRS1 was significantly down-regulated and p-AKT was significantly elevated compared to the PSD group (**Figures 7A, C, D, F**, *P* < 0.01). These results indicate that ZBPYR ameliorate cerebral insulin resistance after chronic psychological stress.

GSK3*β* was a well-known down-stream target of insulin signaling, and we tested the expression of GSK3*β* in ZDF rats. There was no change in the total level of GSK3*β* in the mPFC. However, the p-GSK3*β* expression level was down-regulated in the PSD group, and ZBPYR increased p-GSK3*β* levels compared with PSD group (**Figures 7G–I**, *P* < 0.01).

### Impact of ZBPYR on the mTOR-Autophagy Pathway

**Figure 8:**
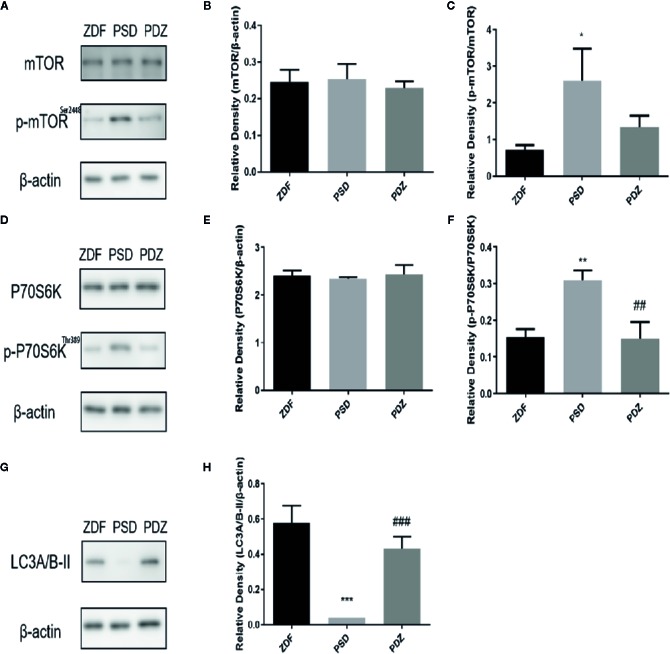
Effect of ZBPYR on the mTOR-autophagy pathway in the mPFC of ZDF rats. Protein expression of mTOR and p-mTOR in the mPFC of each group **(A–C)**. Protein expression of P70S6K and p-P70S6K in the mPFC of each group **(D–F)**. Protein expression of LC3A/B in the mPFC of each group **(G–H)**. Bars represent the mean ± SEM. ^*^ *P* < 0.05, ^**^ *P* < 0.01, ^***^ *P* < 0.001, PSD group *vs.* ZDF group; ^##^ *P* < 0.01, ^###^ *P* < 0.001, PDZ group *vs.* PSD group.

The mammalian target of rapamycin (mTOR) was an atypical serine/threonine protein kinase that regulated autophagy. mTOR was mainly regulated by IRS1/Akt/mTOR signaling pathway, which played important roles in inhibition of cell apoptosis, promotion of cell proliferation, cell survival, and enhancing cognition. Given the involvement of mTOR -autophagy pathway in the clearance of A*β*, we evaluated the molecular mechanism of chronic psychological stress affected cognitive impairments. As expected, a significant increase in the expression of p-mTOR and p-P70S6K was seen in the PSD group (**Figures 8A, C, D, F**, *P* < 0.01), whereas a decrease in the expression of the autophagosome marker LC3A/B was noted (**Figures 8G, H**, *P*<0.001). ZBPYR treatment inhibited phosphorylation of mTOR and P70S6K and enhanced the expression of LC3A/B. There were no significant changes in total levels of mTOR and P70S6K before and after ZBPYR treatment in the three groups (**Figures 8B, E**).

## Discussion

Based on the rapidly increasing prevalence of T2DM and its potential relationship to cognitive decline and dementia, understanding how chronic psychological stress contributes to DACD is of critical public health importance. This understanding is necessary to determine appropriate clinical interventions that will mitigate the risk for cognitive decline in this population. The high efficiency and low toxicity of traditional Chinese medicine (TCM), which has the advantage of providing multiple therapeutic effects on multiple targets, suggest the feasibility of treatment for DACD. ZBPYR, a traditional Chinese medicine, has been shown to be effective for the treatment of T2DM and DACD according to our previous studies (23, 24). The individual herbs used in ZBPYR have different pharmacologic effects, and some of these compounds may contribute to the effects of ZBPYR in preventing or treating diabetes or providing neuroprotection (25–28). However, the mechanism of ZBPYR in regulating DACD induced by chronic psychological stress is unknown.

Stress refers to a physiological and psychological response to environmental changes and noxious stimuli. Compared with acute stress, chronic psychological stress mainly affects the central sympathetic system (norepinephrine system), and continually and repeatedly destroys homeostasis *via* psychological and physiological reactions to environmental changes and regulation of emotions (29). During chronic psychological stress responses, the hypothalamus-pituitary-adrenal (HPA) axis is activated through the secretion of corticotropin-releasing hormone (CRH) and vasopressin from the hypothalamus. The subsequent release of adrenocorticotropin (ACTH) hormone from the pituitary gland initiates glucocorticoid secretion from the adrenal cortex (30). The major glucocorticoid is cortisol in humans and CORT in rodents. CORT can feed back to the brain and affect subsequent behavioral changes (31). Exogenous glucocorticoids also increase human habits-based learning and memory (32). In vitro experiments have also confirmed that elevated CORT can change neuronal cell function and excitability (33). It is believed that CORT is the most important regulator of stress response, as a key node involved in glucose metabolism of T2DM individuals and regulates islet function, and participates in the negative feedback regulation of HPA axis (34). Therefore, we chose CORT as a functional indicator for HPA axis detection. The activity of the HPA axis, a marker of stress, was increased in the present study, as shown by elevated plasma CORT levels. This finding implies that the procedures used to induce chronic psychological stress in this study were enough to cause anxiety. The mPFC, one of the most affected regions in the brain, modulates the expression of emotion processing (35, 36). Moreover, memory-related brain circuits in the mPFC were shown to be impaired in T2DM and prefrontal atrophy who had been diagnosed (37, 38). According to one study, insulin action in the brain, especially in the mPFC, is selectively impaired in T2DM (39). In the present study, mPFC changed synchronously with chronic psychological stress in this study, which indicates a connection between the stress-related cognitive circuits in this region.

A*β* was reported to be the primary pathogenic factor of AD, and A*β*42 deposition is more obvious in the brain of subjects with cognitive decline (40, 41). Relatively small changes in brain A*β* levels early in life appear to be sufficient to lead to early AD pathology in humans, suggesting that an increase in brain A*β* levels over several years in patients with T2DM may lead to cognitive decline (42, 43). Lesions in the mPFC associated with chronic psychological stress could be attributed to the generation of some signaling pathway, such as impaired A*β* plaque and insulin receptor signaling. The APP gene is best known as the precursor molecule whose proteolysis generates A*β* (44). It was reasonable that we observed incremental increases in levels of A*β* and APP in the mPFC after chronic psychological stress because these types of increases also appeared in monkeys with T2DM (45). The present study showed that A*β* levels significantly increased after chronic psychological stress. One potential mechanism is that the chronic psychological stress experienced by rats in this study increased APP levels *via* the inhibition of APP degradation, thus promoting A*β* generation. ZBPYR decreased A*β* and APP levels, thereby partially improving cognitive function. Unfortunately, we have not been able to detect APP hydrolysis intermediates and secretases, though this may be important to explain the underlying mechanisms of changes in terms of A*β* and APP levels, which will be explored in the future as we continue our in-depth research.

The insulin signaling pathway is considered as the major mechanism that involved in the pathogenesis of T2DM and DACD. Recent epidemiologic evidence suggests that CNS insulin resistance is a risk factor for cognitive decline (46, 47). Downstream targets of the insulin signaling pathway, for example, insulin receptor substrate serine phosphorylation 1, were up-regulated in the brains of T2DM rats (48). AKT, a key marker protein of insulin signaling, mediates the effect of insulin *via* important intracellular signaling cascades including the IRS-1/AKT pathway (49). In the study, we identified an increase in p-IRS1 and a decrease in p-AKT in the mPFC of rats undergoing chronic psychological stress, suggesting that central insulin signaling was impaired. ZBPYR may correct CNS insulin resistance by regulating p-IRS1 and p-AKT. In addition, GSK3*β* is a downstream substrate of AKT and is the bridge connecting insulin signaling and A*β* (39, 50). This study found that GSK3*β* activity was improved after ZBPYR treatment, indicating that altered GSK3*β* was mediated by reduced signaling in the IRS-1/AKT pathway. Moreover, our results indicate that the changes in the brains that are consistent with changes in insulin signaling, as well as changes in A*β* deposition, are likely to contribute to the risk of developing AD pathology and clinical manifestations of the disease over time.

In fact, it is well recognized that mTOR signaling is associated with T2DM and AD pathology in several ways, suggesting its potential role in energy imbalance, learning and memory impairment, and A*β* deposition (51, 52). The upstream signal components, which can interact and modulate the mTOR activity, are phosphoinositide 3-kinase (PI3K)/AKT signaling pathways and glycogen synthase kinase 3 (GSK-3) (53). The major downstream target for mTOR is phosphorylate ribosomal protein S6 kinase (P70S6K). Its phosphorylation level is the parameter used to evaluate mTOR activity (54). Our study showed that chronic psychological stress is able to regulate the P70S6K activation response *via* the mTOR pathway, particularly in the brain of ZDF rats. In addition, autophagy is an intracellular degradation process, which is essential for cell growth, survival, differentiation, development, and protein homeostasis. A number of mTOR-autophagy modulators have been shown to have positive effects on cognitive decline. The mTOR inhibition triggers autophagy to decrease A*β* and improve T2DM and AD memory impairment (55). LC3, which is associated with the autophagosome from its formation to its maturation into autolysosome, serves as a marker for autophagy (56). LC3-II is the critical form that needs to be focused on. The present study demonstrated that the expression of phosphorylated mTOR in the cortex region after chronic psychological stress was reduced by ZBPYR treatment, which may be one reason ZBPYR decreased the accumulation of A*β* in ZDF rats. Combined with our *in vitro* research results (data not shown), we speculate that the regulation effect of ZBPYR on chronic psychological stress may be achieved by inhibiting mTOR up-regulation, enhancing autophagy, and then promoting A*β* clearance. Taken together, the present findings provide molecular biological evidence for the preventive effects of ZBPYR on DACD caused by chronic psychological stress in rats.

## Conclusion

In summary, we describe a novel relationship between insulin signaling and A*β* production and accumulation in ZDF rats after chronic psychological stress. Our data suggest that interventions that regulate insulin signaling and A*β* production and accumulation in the brain may provide novel opportunities for treating DACD induced by chronic psychological stress. However, unfortunately, the lack of the negative control group was included to analyze the specific correlation between chronic psychological stress and T2DM disease progression. In particular, future studies should include negative control studies for a better explanation the effects of chronic psychological stress and the role of ZPBYR. In addition, further human studies are required to determine whether A*β* level changes or other variations in the brain could affect insulin secretion or insulin effects, and whether the insulin signaling function in the CNS could modulate insulin effectiveness in peripheral tissues.

## Data Availability Statement

All datasets generated for this study are included in the article/supplementary material.

## Ethics Statement

The animal study was reviewed and approved by the Animal Ethics Committee of Nanjing University of Chinese Medicine.

## Author Contributions

LZ conceived the idea, directed the project, designed the experiments, and involved in modifying the manuscript. TB, WZ, and HS performed the experiments, obtained the samples, and acquired the data. TB conducted the statistical analysis and wrote the manuscript. LZ, WZ, and HS edited the manuscript. All authors contributed to and had approved the final manuscript.

## Funding

This work was supported by the Key Project of the National Natural Science Foundation of China (81230084 and 81730111), a Project Funded by the Priority Academic Program Development of Jiangsu Higher Education Institutions (Integration of Chinese and Western Medicine) and the Postgraduate Research & Practice Innovation Program of Jiangsu Province (No. SJKY19_1412).

## Conflict of Interest

The authors declare that the research was conducted in the absence of any commercial or financial relationships that could be construed as a potential conflict of interest.
